# Power Consumption and Calculation Requirement Analysis of AES for WSN IoT

**DOI:** 10.3390/s18061675

**Published:** 2018-05-23

**Authors:** Chung-Wen Hung, Wen-Ting Hsu

**Affiliations:** Department of Electrical Engineering, National Yunlin University of Science & Technology, 123 University Road, Section 3, Douliou 64002, Yunlin, Taiwan; b10300058@yuntech.edu.tw

**Keywords:** power consumption, Internet of Things (IoT), Advanced Encryption Standard (AES), Electronic Codebook Mode (ECB), Counter with CBC-MAC (CCM), Message Integrity Check (MIC)

## Abstract

Because of the ubiquity of Internet of Things (IoT) devices, the power consumption and security of IoT systems have become very important issues. Advanced Encryption Standard (AES) is a block cipher algorithm is commonly used in IoT devices. In this paper, the power consumption and cryptographic calculation requirement for different payload lengths and AES encryption types are analyzed. These types include software-based AES-CB, hardware-based AES-ECB (Electronic Codebook Mode), and hardware-based AES-CCM (Counter with CBC-MAC Mode). The calculation requirement and power consumption for these AES encryption types are measured on the Texas Instruments LAUNCHXL-CC1310 platform. The experimental results show that the hardware-based AES performs better than the software-based AES in terms of power consumption and calculation cycle requirements. In addition, in terms of AES mode selection, the AES-CCM-MIC64 mode may be a better choice if the IoT device is considering security, encryption calculation requirement, and low power consumption at the same time. However, if the IoT device is pursuing lower power and the payload length is generally less than 16 bytes, then AES-ECB could be considered.

## 1. Introduction

Internet of Things (IoT) devices are becoming more and more popular, and most Wireless Sensor Networks (WSN) IoT devices are usually powered by batteries because the application locations, for example, harsh industrial environments, cannot easily access an electrical grid. Therefore, the power consumption of IoT devices is regarded as an important issue. For example, [1] discussed how to extend the battery life of WSN IoT devices to ten years. In addition, because of the rapid increase in the number of IoT devices in recent years, their number will far exceed the total number of personal computers and mobile phones. If an IoT device does not have encryption, besides the possibility that the data could be stolen, the device may be hacked and controlled, and may even become a botnet. As described in [2,3,4], the security and encryption of the IoT transmission process is very important.

This paper will focus on the analysis of the encryption calculation requirement and power consumption in different payload lengths and AES encryption types, including software-based AES-ECB (Electronic Codebook Mode), hardware-based AES-ECB, and hardware-based AES-CCM (Counter with CBC-MAC Mode). Advanced Encryption Standard (AES) is a widely used block cipher algorithm [5], and it is also one of the encryption methods most commonly used in IoT devices. Note that AES is a symmetric encryption, and its computational complexity is relatively small compared with other asymmetric encryptions. Therefore, it may be more suitable for IoT devices to have only weak computing capability [6,7,8]. However, the AES algorithm still consumes many Central Processing Unit (CPU) cycles, an action that may lead to undesirably large power consumption. As a remedy, hardware implementations are used in many applications, especially in embedded systems, such as [9,10,11]. Previously, it was shown that if S-Box, Shift Rows and other AES matrixes are stored in hardware, the power consumption of circuits can be greatly reduced [12,13,14]. The AES performance comparison between hardware and software is discussed in [15,16].

In order to determine the effects of different AES encryption types on the power consumption and calculation requirement of IoT devices, this paper adopts LAUNCHXL-CC1310 as an experimental platform, which has an ARM Cortex-M3 processor and low-power radio frequency (RF) core. Through the time interval and current of the RF turn-on period, the power consumption of various AES encryption types is measured and analyzed at different data lengths. 

The organization of this paper is as follows. Section 2 introduces the operating principle of each AES mode, and its advantages and disadvantages. Next, Section 3 details the experimental platform and the experimental flow, and analysis of the packet transmission efficiency in different encryption types. Section 4 discusses the encryption calculation requirement and packet transmission efficiency in different encryption types, and then analyzes the overall power consumption. Finally, the conclusions are presented in Section 5.

## 2. Advanced Encryption Standard Details

### 2.1. Advanced Encryption Standard (AES)

AES is a cryptographic algorithm defined by the National Institute of Standards and Technology (NIST) in 2001, known as the Federal Information Processing Standard (FIPS) 197 [17]. It is used by US federal government agencies and other government organizations to protect sensitive electronic data. It is a symmetric block cipher algorithm developed from the Rijndael method [18] and is used to encrypt and decrypt information using the same key. Unlike Rijndael, which handles greater block sizes and key lengths, AES can encrypt and decrypt only 128-bit blocks of data with 128, 192, or 256-bit keys.

### 2.2. Electronic Codebook Mode (ECB)

**Figure 1:**
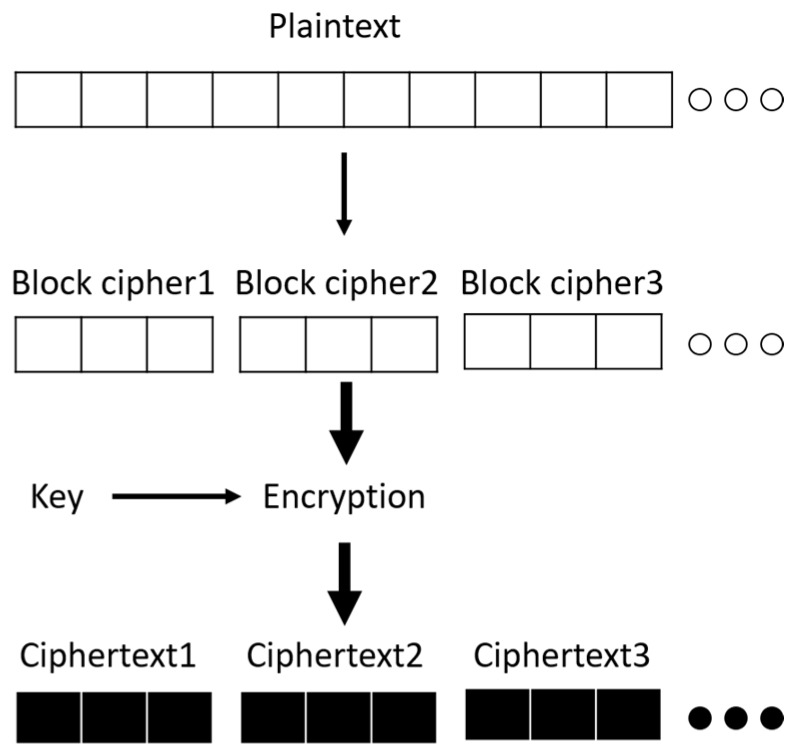
Operation diagram of ECB mode.

The ECB mode [19] is the simplest encryption mode in AES. As shown in Figure 1, the plaintext is divided into multiple blocks, and each block’s size is 16 bytes (128-bit). Then, each block can be independently encrypted in ECB mode. The advantage of ECB mode is its low encryption complexity, but its disadvantage is its lack of random encryption.

### 2.3. Counter with CBC-MAC Mode (CCM)

CCM mode provides data authentication and confidentiality through a combination of counter (CTR) mode and cipher block chaining with message authentication code (CBC-MAC) mode [19]. The CTR and Cipher Block Chaining (CBC) modes will be briefly introduced below.

**Figure 2:**
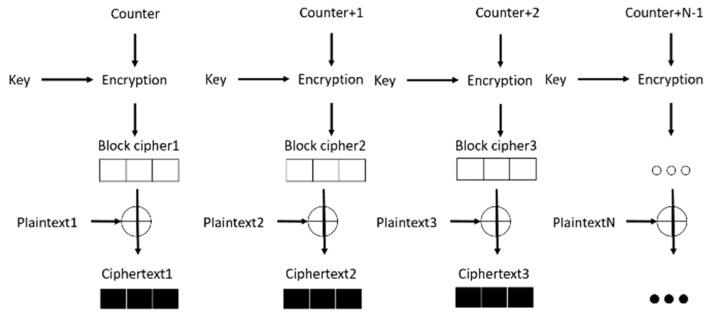
Operation diagram of counter (CTR) mode.

CTR mode is an operating mode that can be used in symmetric cipher algorithms such as AES. As shown in Figure 2, the CTR mode applies the same key to obtain the confidentiality of blocks, and this key can prevent the mode from being identified by an intruder. For each 128-bit block, the values of counter block, called Nonce, must differ from each other. After encryption of the Nonce with a cipher key, a random block cipher will be generated. Then, the block cipher Exclusive-ORs (XORs) the corresponding plaintext block to obtain a ciphertext block. For the last block, which may be a partial block, only the most significant bits associated with the partial block length are used, and the other bits are discarded. Different blocks of CTR can be performed in parallel. CTR mode is used to encrypt data in CCM mode, and the Nonce will increase the randomness of the output ciphertext.

**Figure 3:**
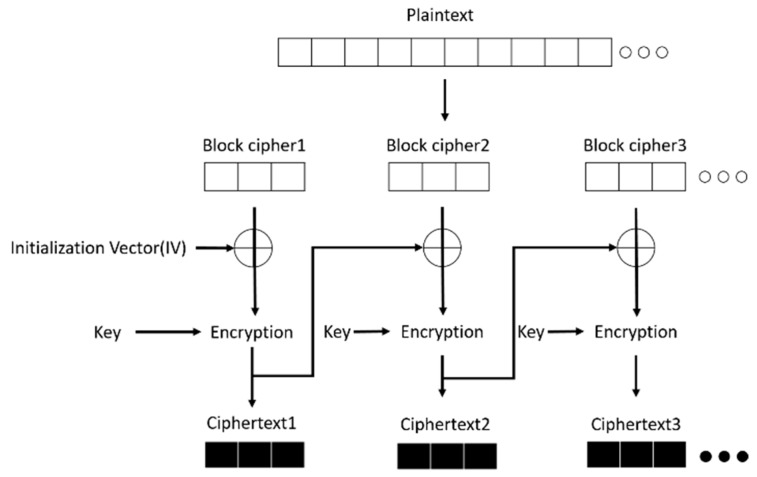
Operation diagram of CBC mode.

CBC-MAC is a process used to generate a message authentication code (MAC) using a block cipher in CBC operation mode. Figure 3 shows the CBC-MAC mode operation. The plaintext data to be verified is encrypted with a deterministic initialization vector in CBC mode, and the deterministic initialization vector is zero. Then, the plaintext data is divided into multiple blocks. The first plaintext block XORs the initialization vector before being encrypted by a key. Next, the result XORs the second message and is encrypted again by the same key. Some of the leftmost bits in the last message block are called authentication tags. The authentication tags are used in the message integrity check (MIC) of the CCM mode.

## 3. Implementation Method

### 3.1. Implementation Platform and Experimental Details

The implementation platform used in this paper was Texas Instruments LAUNCHXL-CC1310. CC1310 is a low-power wireless microcontroller (MCU) embedded with a sub-1G Hz RF core and an AES hardware peripheral. Its ARM Cortex-M3 processor can perform up to 48MIPS, and the RF receiver supports multiple physical layers and RF standards. 

In this paper, the power consumption of different encryption types was measured by fixed LAUNCHXL-CC1310’s RF and AES hardware. There are two sets of LAUNCHXL-CC1310. The one containing the RF transmitter encrypts data before transmission, and the one equipped with the RF receiver decrypts data after receiving it. Moreover, the time-division multiple access (TDMA) mechanism was used in this experiment. In TDMA, the RF transmitter and receiver exchange data at a certain time interval in the cycle. Since the RF receiver must be turned on earlier than the RF transmitter, the RF receiver is not suitable for accurate measurement with the RF turn-on interval, and therefore, the RF current consumption and turn-on interval will mainly focus on RF transmitters.

During measurement, the control variables were 915 MHz for the RF, 200 kbps for the radio data rate, and transmission with a fixed power. The independent variables were different encryption types and different payload lengths. Encryption types included non-encryption, AES-ECB software encryption, AES-ECB hardware encryption, and AES-CCM hardware encryption. The plaintext (payload) lengths included 5 bytes, 20 bytes, 50 bytes, and 100 bytes. Finally, the dependent variables were the RF transmission interval, the AES encryption requirement time interval, and power consumption.

### 3.2. Flow of Different Encryption Types

#### 3.2.1. Non-AES

Non-AES indicates a non-encryption control group. The RF transmitter randomly sends different lengths of data to the RF receiver, and the RF transmission time interval and RF current consumption is measured. In the current experiment, these values were used as the control variables.

#### 3.2.2. AES-ECB-SW

AES-ECB-SW denotes AES-ECB software encryption, and it was used as an experimental group. When using C language to implement software ECB encryption and decryption, the S-Box and Shift Rows matrix need to be initialized, and then the 128-bit key needs to be expanded to the 16 × (10 + 1) bytes of the expanded key. The ECB encryption function is performed before transmission, and the decryption is carried out after the RF receiver receives the encrypted data. In the experiment, the transmission time interval and current consumption of the RF transmitter were measured, and the AES-ECB software encryption calculation resource was also evaluated.

#### 3.2.3. AES-ECB-HW

AES-ECB-HW refers to AES-ECB hardware encryption, and it also was an experimental group. ECB encryption and decryption operations were performed by the AES hardware peripheral on LAUNCHXL-CC1310. In contrast with AES-ECB software, AES-ECB hardware has dedicated read-only memory (ROM) to store S-Box, Shift Rows and other matrixes, and therefore, the memory size requirement of the MCU is significantly reduced. The 128-bit key was initialized and stored in AES hardware before use. The RF transmitter was used to wake up the AES for ECB encryption before the encrypted data transmission. The RF receiver decryptes the payload after receiving it, and the RF transmission time interval, AES encryption time delay required by ECB hardware and the power consumption of AES hardware were measured.

#### 3.2.4. AES-CCM-HW

Similar to AES-ECB-HW, AES-CCM-HW was also an experimental group. The CCM encryption and decryption were performed by the AES hardware that was peripheral on LAUNCHXL-CC1310. Compared to the ECB mode, the CCM mode has an initial Nonce vector and MAC. Nonce can be used to prevent replay attacks, and MAC can be used to check data integrity without tampering. In LAUNCHXL-CC1310, the size of Nonce is adjustable from 7 to 13 bytes. In the proposed experiment, Nonce was set to 13 bytes, and the size value of MAC was set to 8 or 16 bytes. The security levels represented by different MIC lengths we are defined, as shown in Table 1, as levels four to seven, the higher the better [20].

In the experimental item AES-CCM-HW, the RF transmitter and RF receiver were used for the same initial Nonce. The RF transmitter woke up the peripheral AES hardware to perform CCM encryption. The RF payload was transmitted after an additional 8 or 16 bytes of MAC had finished. Then, the RF receiver decrypted the payload and performed the MIC. The RF transmission time interval, encryption calculation requirement, and power consumption were measured.

### 3.3. Analysis of Extra Waste Rate in Different Encryption Types

First, the change in the packet length after different encryption types in different plaintext lengths was analyzed, and the extra waste rate was calculated, as given in (1):

(1) $$\mathrm{Extra}\;\mathrm{waste}\;\mathrm{rate}=\frac{\left(\mathrm{ciphertext}\;\mathrm{length}\;-\;\mathrm{plaintext}\;\mathrm{length}\right)}{\mathrm{ciphertext}\;\mathrm{length}}$$

**Figure 4:**
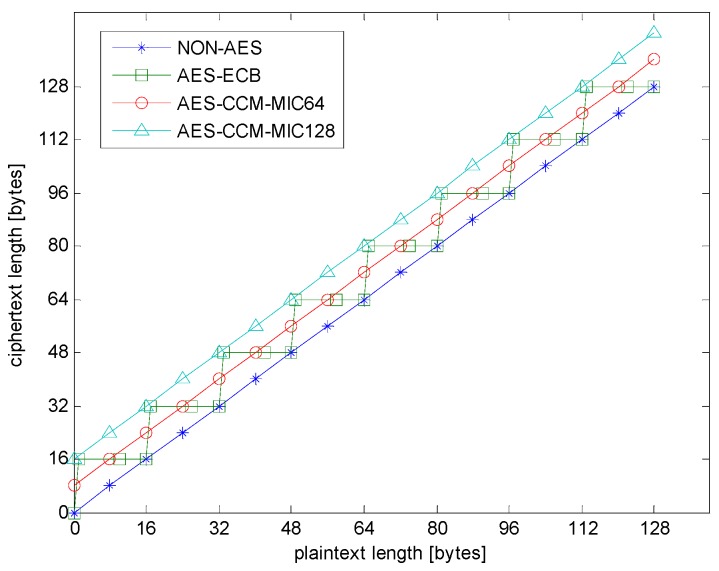
Change in payload length after encryption.

Figure 4 presents the packet length change after the encryption of AES-ECB and AES-CCM. The *X* axis represents the payload length before encryption, and the *Y* axis represents the encrypted data length after encryption. Note that non-AES is the experimental control group without any AES encryption, so its *X*-axis value always equals that of the *Y*-axis. The block unit of the AES-ECB was 16 bytes. When the length of payload in the block was less than 16 bytes, the other bytes were filled with zeros, and its ciphertext length was always a multiple of 16 bytes. For AES-CCM, the ciphertext length was always longer than or equal to the plaintext length, and the additional length was the length of MAC, which was 8 or 16 bytes.

**Figure 5:**
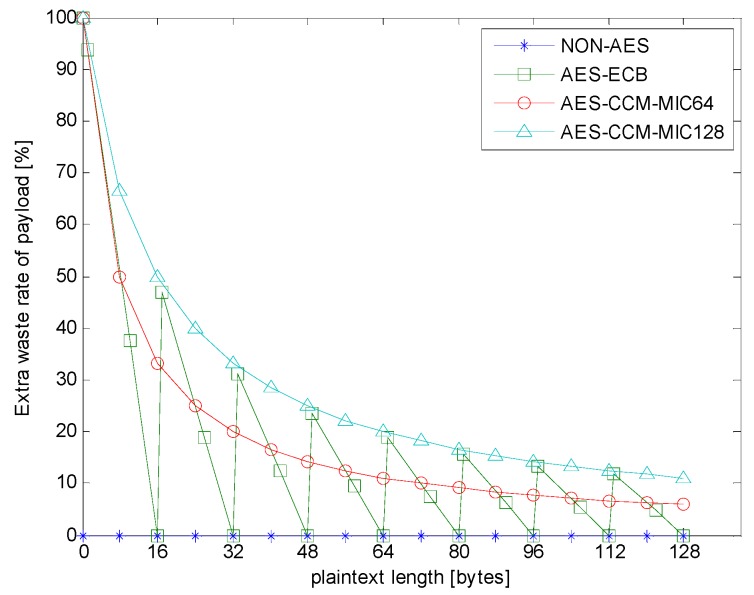
Extra payload waste rate of encryption.

Figure 5 presents the extra waste rate in different encryption types. First, non-AES had no encryption calculation, so the extra waste rate was always zero. As shown in Figure 5, in AES-ECB and AES-CCM, when the payload length was less than eight bytes, the extra waste rate exceeded 50 percent. The extra waste rate of AES-ECB changed drastically when the payload length neared multiples of 16 bytes. Compared with AES-ECB, the extra waste rate of AES-CCM decreased steadily towards a constant value.

Obviously, the extra waste rate of AES-ECB decreased more quickly than other that of other types when the plaintext length was less than 16 bytes. However, as the plaintext length increased, this advantage of AES-ECB gradually reduced. Moreover, AES-CCM had higher security because of Nonce, and AES-CCM output different ciphertext when the plaintext was repeated. Therefore, if the plaintext exceeds a certain length, AES-CCM is theoretically more competitive.

## 4. Experimental Results and Analysis

### 4.1. Encryption Power Consumption of Different Encryption Types

For AES hardware, the encryption of one block of data required approximately 32 clock cycles [21]. If the CC1310 MCU runs at 48 MHz, a single encryption operation runs in approximately 0.667 μs. However, the calculation of software operation is necessary, including the AES hardware API and other software encryption processes. The details and analysis will be discussed later.

The CC1310 MCU ran normally under sleep mode in this experiment, and the current was about 0.836μA. When the AES hardware encryption was used, not only hardware operations, but also software API calls and other software processes were required. These actions, including both the hardware and software operations, consumed much more current than sleep mode. Therefore, the following experimental data mainly refers to the total time of AES hardware and software operations, as well as the average current consumption during this period.

Table 2 shows the average encryption time interval for the four encryption types: AES-ECB-SW, AES-ECB-HW, AES-CCM-HW-MIC64, and AES-CCM-HW-MIC128. AES-ECB-SW is the software AES-ECB operation, so its average encryption time was much longer than the other two. AES-ECB-HW is a hardware AES-ECB operation, but the hardware can only handle 16 bytes of plaintext at a time. Therefore, when the length exceeded 16 bytes, some extra software processing was required, leading to a longer processing time. Finally, AES-CCM-HW is mainly hardware AES-CCM operation, and its software only took a few clock cycles to call the API without 16-byte-plaintext limitation.

Table 3 shows the average current consumption in five encryption types: non-AES, AES-ECB-SW, AES-ECB-HW, AES-CCM-HW-MIC64, and AES-CCM-HW-MIC128. Non-AES is the control group, and the average current consumption only represents the movement of data to the RF module. The other four average current consumptions represent the sum of encrypting and transferring data.

After multiple current measurements, the average currents of AES-ECB-HW and AES-CCM-HW were more than that of non-AES by about 1.14 mA, and the additional current was consumed by AES hardware. The current consumptions of AES-ECB-SW and non-AES were similar. Therefore, the main difference in power consumption depends on whether AES hardware is working.

**Figure 6:**
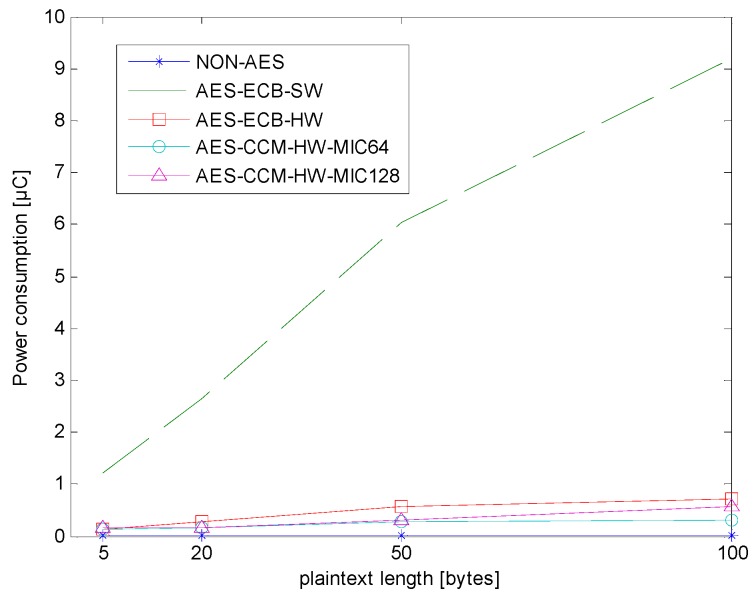
Encryption power consumption of different encryption types.

Figure 6 shows the power consumption of different encryption types in different plaintext lengths. Again, non-AES is an experimental control group. In non-AES, the time for only moving data for the RF module was quite short, less than 1 μs, and the power consumption was minimal. The AES-ECB-SW had the lowest current in Table 3, but the longest processing time in Table 2. Hence, its power consumption was the highest. Additionally, in the case of a longer payload length, AES-CCM-HW will consume less power than AES-ECB-HW.

### 4.2. Transmission Power Consumption of Different Encryption Types

When in RF transmission, the AES hardware was turned off. The RF average transmission currents of non-AES, AES-ECB-SW, AES-ECB-HW, AES-CCM-HW-MIC64, and AES-CCM-HW-MIC128 were similar, and the values were about 28.2361 mA. The main difference between the different encryption types was the encrypted data length, which is the main reason for the increase in transmission power consumption. Therefore, this experiment will measure the transmission time affected by the extra data length.

In the experiment, the RF transmission data rate was set to 200 kbps, so, transmitting one byte took about 40 μs. In addition, the CC1310 basic packet header was 10 bytes, including four preamble bytes, four sync word bytes, one length byte, and one address byte. Every packet also required four CRC bytes. Therefore, each payload had an extra transmission time of about 560 μs.

Table 4 shows the average transmission times of encrypted packets in different plaintext lengths and different encryption types. Using a digital-oscilloscope, one can measure the average transmission time in the duration of the non-zero RF transmission current. Non-AES was the control group, and AES-ECB-SW, AES-ECB-HW, AES-CCM- HW-MIC64, and AES-CCM-HW-MIC128 were experimental groups. The data in Table 4 can be compared with the trends shown in Figure 1 and Figure 2. As expected, the measured results for all four cases show that the transmission time increased with the plaintext length and was very close to the time obtained by multiplying 5 μs, the one-bit period of the 200 kbps transmission data rate, with the number of bits involved.

**Figure 7:**
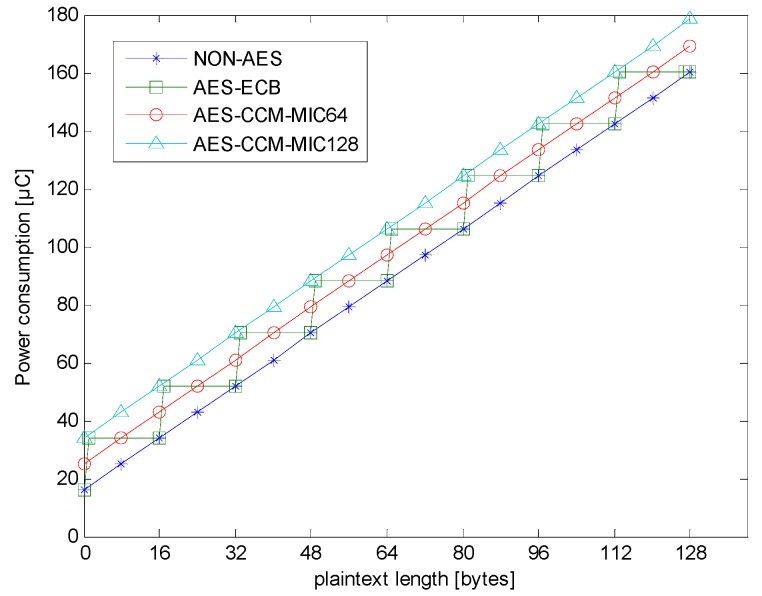
Transmission power consumption of different encryption types.

Figure 7 shows the power consumption of the transmission. It can be seen that the overall power was mainly affected by the payload length. Therefore, the trend of Figure 7 is similar to Figure 1. There was no difference in transmission time between AES-ECB-SW and AES-ECB-HW, so their transmission power consumptions were the same.

The transmission data rate of RF was also one of the main factors affecting the power consumption. This is because a slower rate corresponds to a longer transmission time, and thus a higher power consumption. Conversely, if the transmission rate becomes faster, the power consumption will become smaller. Since this experiment mainly analyzed the power change caused by AES encryption, the transmission data rate is not detailed here.

### 4.3. Analysis of the Total Power Consumption of Different Encryption Types

In this section, data from the analysis of the total power consumption and encryption security in different encryption types is reported. In addition, the issue regarding which encryption type is the most power-saving or most suitable in different payload lengths is discussed.

**Figure 8:**
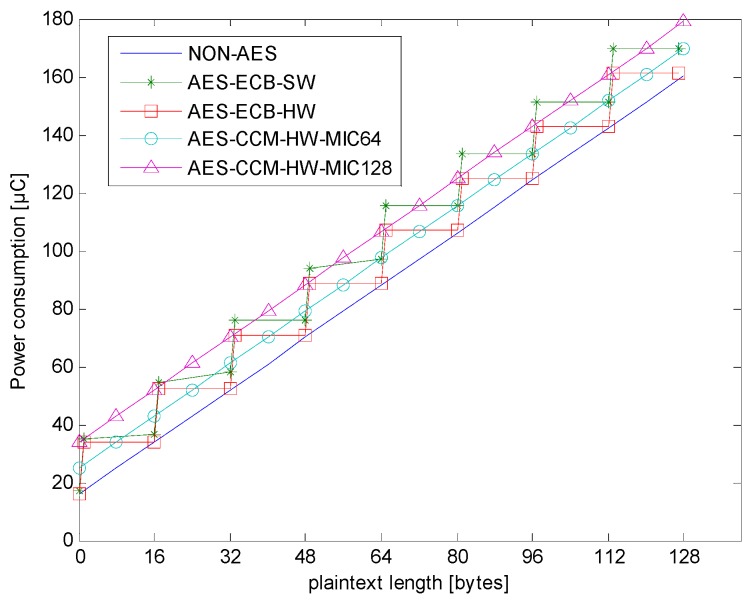
Total power consumption of different encryption types.

Figure 8 shows the total power consumption of encryption operations and transmissions. There was a large correlation between power consumption and payload length. Only AES-ECB-SW had a large amount of encryption power consumption, and most of the power consumption in other types were mainly due to transmission.

**Figure 9:**
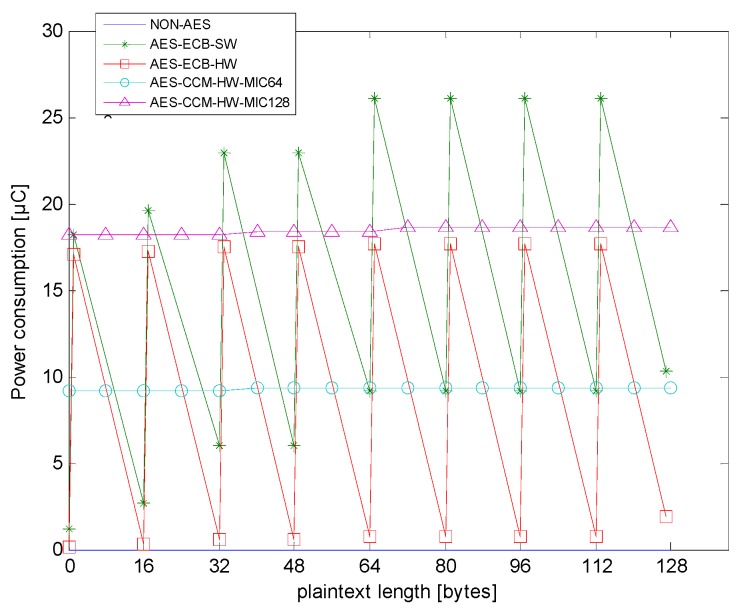
Additional power consumption of different encryption types.

Figure 9 shows the additional power consumption of different encryption types. The additional power consumption is the difference between the total power consumption of AES and that of non-AES.

From Figure 9 and Table 2, one can see that AES-ECB-HW performs better than AES-ECB-SW in terms of power consumption and encryption calculation requirements. In the AES hardware encryption type, the average power consumptions (shown in Figure 9) were 9.0884 μC for AES-ECB-HW, 9.3626 μC for AES-CCM-HW-MIC64, and 18.6110 μC for AES-CCM-HW-MIC128. Among them, the average power consumption difference between AES-ECB-HW and AES-CCM-HW-MIC64 was 0.2742 μC, and the power consumption of AES-CCM-HW-MIC128 was larger than those of the other two for any payload length.

## 5. Conclusions

The data in Table 2 confirms that the power and calculation performances of hardware AES are much better than those of software AES. Therefore, hardware AES is the better choice to allow low power and high calculation performances.

In the three AES hardware methods, ECB, CCM-MIC64, and CCM-MIC128, the CCM-MIC128 mode showed the highest level of security sequencing, followed by the CCM-MIC64 and then the ECB mode. On the other hand, the ECB mode had the lowest total power consumption, the CCM-MIC64 mode the second, and the CCM-MIC128 mode the highest. In terms of encryption calculation requirement, the ECB mode performed slightly better when the payload length was less than 16 bytes, and the CCM mode performed better for other lengths.

In summary, AES-CCM-HW-MIC64 is a good choice when considering security, encryption calculation requirement, and low power consumption together. However, if the IoT device in question must pursue the lowest power consumption with a payload length that is not long, then AES-ECB may be considered.

## Figures and Tables

**Table 1:** Security levels provided by the IEEE802.15.4 Spec.

Security Attributes	Authentication Tag Length (Bytes)	Security Level
Encryption (ENC)	0	4
ENC-MIC-32	4	5
ENC-MIC-64	8	6
ENC-MIC-128	16	7

**Table 2:** Average encryption times of different encryption types in different payload lengths.

Plaintext Length (Bytes)	Encryption Type			
	AES-ECB-SW	AES-ECB-HW	AES-CCM-HW-MIC64	AES-CCM-HW-MIC128
5 bytes	366.27 μs	30.29 μs	30.49 μs	31.17 μs
20 bytes	732.51 μs	60.19 μs	31.53 μs	32.09 μs
50 bytes	1464.77 μs	120.66 μs	60.84 μs	62.09 μs
100 bytes	2533.01 μs	152.53 μs	61.61 μs	120.34 μs

**Table 3:** Average current consumption of different encryption types.

Encryption Type	Current Consumption
Non-AES	3.6273 mA
AES-ECB-SW	3.6275 mA
AES-ECB-HW	4.7696 mA
AES-CCM-HW-MIC64	4.7684 mA
AES-CCM-HW-MIC128	4.7686 mA

**Table 4:** Average transmission times of different encryption types in different payload lengths.

Plaintext Length (Bytes)	Encryption Type				
	Non-AES	AES-ECB-SW	AES-ECB-HW	AES-CCM-HW-MIC64	AES-CCM-HW-MIC128
5	758 μs	1203 μs	1196 μs	1081 μs	1402 μs
20	1357 μs	1847 μs	1840 μs	1679 μs	2482 μs
50	2561 μs	3119 μs	3118 μs	2880 μs	3201 μs
100	4567 μs	5681 μs	5678 μs	4881 μs	5219 μs
